# Men with Crohn’s disease may have an increased risk for head and neck squamous cell carcinoma — a nationwide register study

**DOI:** 10.1007/s00784-022-04762-w

**Published:** 2022-10-29

**Authors:** Anni Harjunen, Enna Puolakkainen, Hanna K. Laine, Jaana Rautava

**Affiliations:** 1 Oral Pathology and Oral Radiology, Faculty of Medicine, University of Turku, Turku, Finland; 2 Department of Oral and Maxillofacial Diseases, Clinicum, Faculty of Medicine, University of Helsinki, Box 41, FI-00014 Helsinki, Finland; 3 Department of Pathology, Medicum, Faculty of Medicine, University of Helsinki, Helsinki, Finland; 4 HUS Diagnostic Center, HUSLAB, Helsinki University Hospital, Helsinki, Finland

**Keywords:** Inflammatory bowel disease, Crohn’s disease, Ulcerative colitis, Oral cancer, Head and neck cancer

## Abstract

**Objective:**

Our goal was to study inflammatory bowel disease (IBD) patients’ risk of head and neck squamous cell carcinoma (HNSCC), compared to general population.

**Materials and methods:**

We performed a retrospective nationwide register-based study of Finnish individuals diagnosed with IBD between the years 1995 and 2015. The standardized incidence ratio (SIR) of HNSCC was calculated by comparing the cohort’s complementary age-year-sex-person-year incidence to that of the whole Finnish population.

**Results:**

About 70,567 patients were diagnosed with IBD (Crohn’s disease or ulcerative colitis). Later, 89 of them were diagnosed with HNSCC with mean time of 6.82 years. The incidence of HNSCC was increased in IBD patients compared to the Finnish population expectation (*SIR* 1.3, 95% *CI* 1.065–1.614, *P* = 0.062). When calculating Crohn’s disease and ulcerative colitis separately as well as men and women separately, the incidence was particularly increased for men with Crohn’s disease (*SIR* 1.951, 95% *CI* 1.216–2.935, *P* = 0.025).

**Conclusion:**

An increased risk for HNSCC was found in men with Crohn’s disease compared to the Finnish population expectations.

**Clinical relevance:**

This study provides information that would improve follow-up protocols and treatment guidelines of IBD.

## Introduction

Recently, there have been suggestions that inflammatory bowel diseases (IBD) increase the risk of head and neck squamous cell carcinoma (HNSCC) [1–4]. Squamous cell carcinoma (SCC) is the most common type of head and neck cancer. SCC covers approximately 90% of head and neck cancers [5]. HNSCC is often diagnosed late, and the 5-year survival is only slightly above 50% [6]. Well-established major risk factors for HNSCC are smoking and alcohol use. In addition, eating habits, deficiency diseases, viruses (including human papilloma virus, HPV), and possible genetic susceptibility seem to increase the HNSCC risk [7].

Crohn’s disease can affect any part of the digestive tract, including the mucosa in the oral cavity [8–10]. The oral manifestations of Crohn’s disease include aphthous ulcers, cobble stoning mucosa, cheilitis, swelling of the lips and other ulcers. These manifestations appear in 20 to 50% of the patients at some point in their lives [11]. Ulcerative colitis (UC)-related oral manifestations include superficial ulcerations and pyostomatitis vegetans [2, 12]. The prevalence of oral manifestations in UC patients has been reported to vary from 2 to 34% [13]. These oral lesions often cause discomfort, pain and difficulties in eating and oral hygiene. Oral mucosal lesions of IBD may serve as first signs that lead to diagnosis.

Patients with IBD are at an increased risk of developing cancer [14, 15]. This risk seems to be associated at least partly with immunosuppressive medications [16]. In addition, chronic inflammation (of any kind) itself is known to increase cancer risk [12]. Three studies have suggested that IBD patients are at increased risk of developing HNSCC [3, 4, 17]. The hypothesis of the present study is that individuals with IBD have an increased risk of developing HNSCC compared to general population. If this hypothesis is true, patients with IBD should be considered as HNSCC risk patients.

## Material and methods

This study was approved by Finland’s National Institute for Health and Welfare (THL) (Dnro THL/2220/5.05.00/2017; December 13, 2018) and Statistics Finland for the dates of death (TK-53–503-19; March 29, 2019).

Data (social security number, IBD diagnosis and the year of diagnosis) of the adult (≥ 18 years of age and older) Finnish citizens diagnosed with IBD (ICD-10 codes K50 and K51) between the years 1995 and 2015 were collected from the Care Registers for Social Welfare and Health Care (HILMO) powered by the THL. If the patient had both ICD-10 codes (9310 patients out of 70,567, 13.2%), the patient was considered in the first mentioned IBD cohort. Patients with a head and neck cancer prior to IBD diagnosis were excluded from the study. The collected data were then compared to data from the Finnish Cancer Registry considering head and neck cancers (ICD-O-3 C00-14). After considering all cancers, only squamous cell carcinomas were included in this study. The possible dates of deaths were collected from the Statistics Finland. If the IBD patient had died, the follow-up was considered complete at the time of death.

The standardized incidence ratio (SIR) was calculated by comparing the cohort’s complementary age-year-sex-person-year incidence (per 100,000 person-years) to that of the whole Finnish population. By using this number, the expected number of cancer cases was calculated and compared to the observed number of cancer cases. P-values and confidence intervals for standardized incidence ratios were calculated from profile-likelihood of a Poisson-model. A multiple comparison adjusted *P*-value (Holm’s method) was also calculated for the patients with Crohn’s disease and UC.

## Results

### IBD patients

Altogether, 70,567 patients were diagnosed with IBD between the years 1995 and 2015 in Finland. Crohn’s disease was diagnosed in 19,694 (10,335 women, 9359 men) individuals. UC was diagnosed in 50,873 (24,347 women, 26,526 men) individuals. Age at IBD diagnosis varied from 18 to 91 years; mean age was 46.5 years.

### IBD patients with HNSCC

Of the IBD patients, 89 were later diagnosed with HNSCC (ICD-O-3 C00-14). HNSCC developed in 28 individuals with Crohn’s disease (8 women, 20 men) and in 61 (22 women, 39 men) with UC. Of the HNSCCs, 22 were on the tongue, 19 on the lips, 12 in the tonsillar areas or overlapping sites of tonsils, 10 in the hypo- and oropharynx, 8 on the floor of mouth and 9 in other parts of the mouth (palate, buccal mucosa, gingiva, vestibule of mouth and vallecula). Eight of the HNSCCs occurred in unspecified area of the head and neck. The age of these patients ranged from 36 to 92 years at the time of HNSCC diagnosis; mean age was 66 years. The mean time from IBD diagnosis to HNSCC diagnosis was 6.82 years, and it ranged from 0.33 to 19.66 years.

### IBD and HNSCC risk

**Fig. 1:**
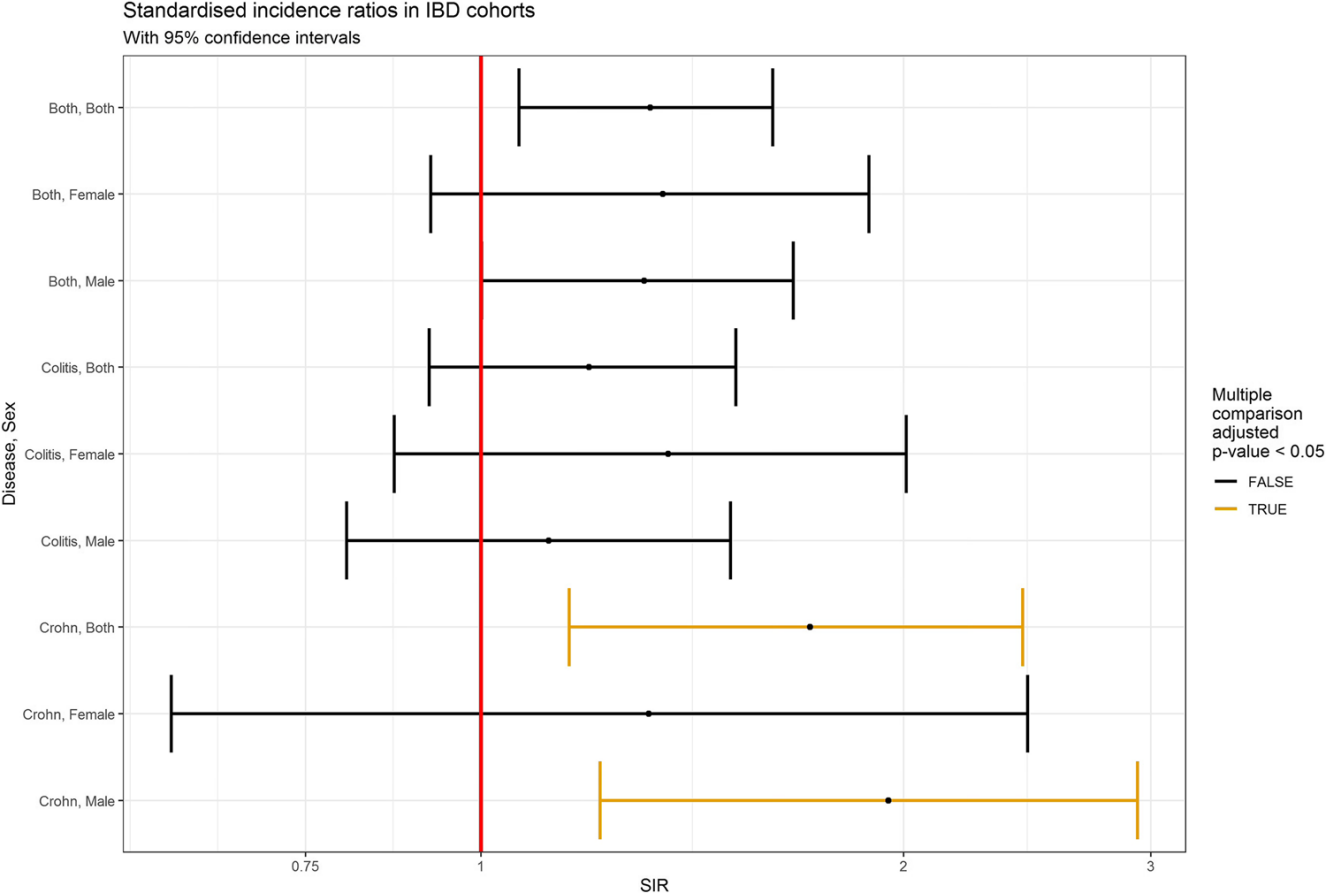
Standardized incidence ratios (SIR) of squamous cell carcinoma in inflammatory bowel disease (IBD) cohorts

According to *SIR* calculations, the incidence of HNSCC was increased in IBD patients compared with that of the Finnish population expectation in general (*SIR* 1.3, 95% *CI* 1.065–1.614, *P* = 0.062) (Table 1) (Fig. 1). However, when Crohn’s disease and UC were calculated separately, only individuals with Crohn’s disease had a statistically significantly increased incidence of HNSCC (*SIR* 1.715, 95% *CI* 1.156–2.431, *P* = 0.034). When calculating men and women with Crohn’s disease separately, the incidence was increased for men with Crohn’s diseases (*SIR* 1.951, 95% *CI* 1.216–2.935, *P* = 0.025) but not for women (*SIR* 1.317, 95% *CI* 0.602–2.451, *P* = 0.873).

**Table 1:** Standardized incidence rations (*SIR*) head and neck squamous cell carcinoma in inflammatory bowel disease cohorts (CU = ulcerative colitis; CD = Crohn’s disease; both = CU and CD)

Disease	Sex	Observed	Expected	pyrs	*SIR*	*SIR*.lo	*SIR*.hi	*P* value	Category	Adjusted *P*
Both	Both	89	67.4156	587,448.14	1.3202	1.0645	1.6137	0.00878	2	0.06146
Both	Male	59	45.1531	294,942.85	1.3067	1.0010	1.6690	0.03992	2	0.23952
Both	Female	30	22.2625	292,505.29	1.3476	0.9211	1.8889	0.10229	2	0.51145
CU	Both	61	51.0888	427,088.30	1.1940	0.9189	1.5192	0.16611	2	0.60044
CU	Male	39	34.9017	220,008.39	1.1174	0.8024	1.5057	0.48808	2	0.87268
CU	Female	22	16.1871	207,079.90	1.3591	0.8673	2.0087	0.15011	2	0.60044
CD	Both	28	16.3268	160,359.84	1.7150	1.1556	2.4309	0.00431	2	0.03448
CD	Male	20	10.2514	74,934.45	1.9509	1.2159	2.9351	0.00290	2	0.02520
CD	Female	8	6.0754	85,425.39	1.3168	0.6018	2.4509	0.43634	2	0.87268

## Discussion

According to our national register study, men with Crohn’s disease were at higher risk of developing HNSCC. This supports the findings of four previous studies that suggest that IBD patients have an increased risk for HNSCC [3, 5, 17]. However, another nationwide registry-based study from Denmark by Pasternak et al. [16] did not find this connection when evaluating IBD patients with azathioprine medication and all cancers. A recent study using Mendelian randomization analysis by Chen and co-workers from China reported that this is true for oral cavity carcinomas, but not oropharyngeal carcinomas [18].

The incidence of IBD is highest in North America and Western Europe and is increasing worldwide [19]. The incidence of IBD in the Finnish population is comparable to the countries in North America and Western Europe. In Finland, the annual prevalence of Crohn’s disease is 9.2/100.00 and UC 24.8/100.00, and the incidence is increasing [20]. In our study with 70,567 patients with IBD, most patients were diagnosed with UC (72%) and about one-third (28%) with Crohn’s disease. Although most of the IBD patients are diagnosed between the ages of 16 and 30 years old, the disease can appear at all ages [21]. In our study, only patients ≥ 18 years were included, and the age at the IBD diagnosis ranged from 18 to 91 years with a mean age of 46.5 years. In the current study, only adult patients (≥ 18 years) were included which explains the discrepancy of the age range described in the literature. With Crohn’s disease, the incidence seems to be similar between men and women; with UC the incidence has been reported to be higher in men [22]. In our cohort, there were no major differences between the sexes. Crohn’s disease had a slight female predominance (52%) while there was a slight male prominence (52%) with UC.

The three previous studies that suggest an increased risk of HNSCC in IBD patients evaluated cohort different than ours. The cohort most similar to ours was examined in the study by van De Ven and co-workers [17] on IBD and laryngeal carcinoma, which was a nationwide registry-based study from Denmark. Nissen et al. [4] performed a case–control study from a Dutch registry. Katsanos et al. [3] studied a cohort from a single IBD referral centre, The Mount Sinai Medical Center. The current Finnish population is very homogenous with mostly Caucasian, Finland-born individuals [23]. Despite the differences between cohorts, our results on HNSCC risk were surprisingly similar to those from the study by Katsanos et al. [3]. Of our 70,567 IBD patients, 89 (0.12%) were diagnosed with HNSCC. In the study of Katsanos et al. study [3], 11 out of 7294 patients (0.15%) were found to have oral cancer. The other two studies did not mention the size of the entire IBD population. A recent study from Chen et al. [18], however, suggested that oropharyngeal carcinoma may differ from oral cavity carcinoma. In our study, the mean time from the IBD-diagnosis to the HNSCC-diagnosis was 6.82 years and ranged from 0.33 to 19.66 years. In the study of Nissen et al. [4], the median time from IBD diagnosis to the HNSCC diagnosis was 9.00 years. Katsanos et al. [3] reported a longer mean time (17.6 years, range 2–40 years) from IBD diagnosis to oral cancer diagnosis. Van de Ven et al. [17] did not report the time between IBD diagnosis and HNSCC diagnosis.

The *SIR*-number is used to clarify the incidence of cancer in certain groups. This number was either higher, equal or smaller than expected. In our study, the *SIR*-number was > 1 in every case (1.1174–1.9509). This means that the incidence of HNSCC was higher than expected in all cases. This is important as we categorized the patients according to their first IBD diagnosis in the system. Therefore, it is possible that there was some fluctuation from CU to Crohn’s disease and vice versa. The *SIR* for HNSCC with Crohn’s disease was 1.7150 (95% *CI*, 1.1556–2.4309) and HNSCC with CU was 1.1940 (95% *CI*, 0.9189–1.5192). However, when calculating men and women with Crohn’s disease separately, the incidence was increased for men with Crohn’s diseases (*SIR* 1.951, 95% *CI* 1.216–2.935, *P* = 0.025) but not for women (*SIR* 1.317, 95% *CI* 0.602–2.451, *P* = 0.873). Our results suggest that the risk of developing HNSCC was particularly increased in men with Crohn’s disease. Finnish men with Crohn’s disease have 2.0 times higher risk of getting HNSCC, compared to the rest of the population. The study by Katsanos et al. [3] revealed that women had higher risk for oral cancer compared to men. They observed that patients with IBD were at an increased risk for oral cancer (*SIR* 9.77, 95% *CI* 5.14–16.98). The age- and sex-adjusted *SIR* for oral cancer in women was 12.07 (95% *CI*, 3.84–29.11) and in men 8.94 (95% *CI*, 3.71–16.78). These results may reflect differences between cohorts and observation periods. However, both studies suggest that IBD increases the risk of developing HNSCC.

It is well established that patients with IBD are at increased risk of developing cancer in the gastrointestinal tract. Patients with Crohn’s disease have a 20- to 30-fold increased risk of cancer in the small intestine, and patients with UC have a higher risk of colorectal cancer [14, 15]. This increased risk may be due to the persistent inflammation and is linked to the duration and extent of disease [24]. Some IBD patients have mucosal lesions in the oral region. In Crohn’s disease granulomatous inflammation associated lesions could be more severe than more erosion type lesions in ulcerative colitis [9]. Biopsies reveal granulomatous inflammation in Crohn’s disease and superficial ulcerations and unspecific inflammation in UC. Patients with IBD-related oral lesions often have such lesions for a short period of time, although some patients have persistent lesions. We have previously shown in an IBD mouse model that colitis that causes colon dysbiosis can also cause dysbiosis in the oral cavity [25], which offers one possible explanation of oral IBD-related lesions. However, Read et al. have proposed that dysbiosis in the oral cavity could escalate IBD via bacteria translocation to intestine [26] and especially periodontitis has been suggested to worsen IBD [27]. In addition, oral microbiota alteration towards dysbiosis has been related to oral cancer development, and on the other hand probiotics may have anti-tumoral activity [28]. None of the studies with IBD and HNSCC, including ours, have information on possible IBD-related oral lesions in these patients. However, the lager amount of HNSCCs within Crohn’s disease patients in our study could be related to more severe inflammation in the oral mucosa. Another possible reason for the increased cancer risk in IBD patients is IBD medications. These medications have immunosuppressive effects, and studies have suggested that IBD treatment modalities are associated with increased oral cancer risk [29–31]. However, if this was the case, one would expect to see this in both sexes. Nevertheless, in our study only the men were at significantly increased risk. One reason could be that men use tobacco when compared with women [32]. According to the Finnish Cancer registry, the incidence of tongue cancer is increasing particularly among men [33]. Furthermore, according to Erol and Karpyak, women are more likely lifetime abstainers or consume less alcohol than men [34] which fits for Finnish population as well [35]. We suggest the possibility that if men with Crohn’s disease have more severe disease than women with Crohn’s disease, this could increase the HNSCC incidence in men. However, at least one study with paediatric patients has shown that girls with Crohn’s disease tend to have more severe disease compared to boys [36]. Our study is register-based, and we do not have information on disease severity or possible HNSCC risk factors.

Aging is another risk factor for HNSCC occurring commonly between 40 and 70 years of age [37]. However, cancer site and other HNSCC risk factors affect as well. According to Ghazawi et al., the incidence of oral cavity SCC increased with age, but oropharyngeal SCC had a stable incidence peak at 50–59 years [38]. In the present study, the mean age when IBD patients were diagnosed with HNSCC was 66 years old (range 36–92 years). In the Katsanos et al. study [3], the mean age at the time of HNSCC diagnosis was 44.6 ± 17.4 years (range 41–81 years). The Nissen et al. study [4] presented a median age when IBD patients developed HNSCC, 60.5 years. Nissen et al. [4] discussed that increasing age at IBD diagnosis was associated with a greater risk of HNSCC. Although our patient cohort was older than those of the other studies, the HNSCC incidence was similar and was closest to the average age of developing HNSCC.

The most common location for any HNSCC is the tongue [39]. In our study, the most common site of HNSCC development in patients with IBD was similarly the tongue (22/89, 25%). Furthermore, in the study of Katsanos et al. [3], the tongue was the most common location for the malignancy. The third most prevalent site for the HNSCC in our study was the tonsillar areas or the overlapping sites of tonsil (12/89, 13%). HPV infection is particularly associated with HNSCC on oropharyngeal regions, especially the tonsillar areas [40]. The study by Katsanos et al. [3] reported that 12/23 cases were HPV positive. The incidence of HPV-related HNSCC is currently increasing [41]. In our study, the data on HNSCC were based on a cancer registry and did not include the information on HPV status. If our results are consistent with those from Katsanos et al. [3], with rather high HPV positivity with oropharyngeal carcinomas, we would estimate that six of our patients most likely had HPV-positive HNSCCs.

Our study has limitations. Since the present study is a retrospective and is based only on Finnish national register databases, it is not possible to verify any data in patient charts. We have no patient-related information on the possible risk factors for HNSCC, such as tobacco, alcohol consumption or HPV-infection. We also did not have information on the patients’ medications (e.g. immunosuppressants) or oral manifestations. Therefore, identifying the drivers for the increased risk of HNSCC requires further studies. Another limitation is that we have not done separation between different locations of head and neck SCC. In the future, it is also important to study head and neck SCCs separately in different locations since they might have differences in the pathogenesis, as suggested by Chen et al. [18]. The strength is that this was a Finnish nationwide and register-based study. In Finland, patients’ medical diagnoses (including IBD diagnoses) from public and private health care are collected in the national files of HILMO, and cancer diagnoses are collected in the Finnish Cancer Registry. THL is an independent research institute working under the Ministry of Social Affairs and Health in Finland. Finnish registries have previously been shown to have an excellent coverage [42, 43]. The coverage and accuracy of the Finnish Cancer Registry data are over 98% of all cancers [44, 45]. Therefore, our cohort is extensive, comprehensive and reliable and should include all adult patients diagnosed with Crohn’s disease or UC between the years 1995 and 2015 in Finland and the HNSCCs they later have developed.

In conclusion, we have shown with the largest ever IBD-HNSCC registry-based cohort which include all Finnish patients with IBD during a period of 20 years that IBD may increase risk for HNSCC. The risk could be particularly increased in men with Crohn’s disease. Based on this finding, regular oral mucosal inspections should be suggested to men with Crohn’s disease. This could reduce any possible delays in HNSCC diagnosis. Early diagnosis of HNSCC substantially increases the overall 5-year survival rate [46].
